# ACTH-Independent Cushing's Syndrome Associated with Left Adrenocortical Oncocytoma of Uncertain Malignant Potential

**DOI:** 10.1155/2020/8816527

**Published:** 2020-09-26

**Authors:** Giuseppe S. Sica, Leandro Siragusa, Bruno Sensi, Vittoria Bellato, Pierangela Floris, Valentina Rovella, Alessandro Mauriello, Monia Di Prete, Rossana Telesca, Valerio Ciavoni, Carmine Cardillo, Nicola Di Daniele, Manfredi Tesauro

**Affiliations:** ^1^Department of Surgery, University of Rome “Tor Vergata”, 00133 Rome, Italy; ^2^Department of Systems Medicine, University of Rome “Tor Vergata”, 00133 Rome, Italy; ^3^Department of Experimental Medicine, University of Rome “Tor Vergata”, 00133 Rome, Italy; ^4^Department of Internal Medicine, Universitá Cattolica del Sacro Cuore, Rome 00168, Italy

## Abstract

Adrenocortical oncocytomas are rare and mostly nonfunctioning neoplasms. We report the case of a 27-year-old woman diagnosed with an ACTH-independent Cushing's syndrome due to left adrenal oncocytoma. She underwent laparoscopic adrenalectomy. Histopathological examination revealed an oncocytoma of uncertain malignant potential with a low Ki-67 proliferation index, inhibin A positivity, and chromogranin A negativity. Electron micrographs confirmed adrenal oncocytoma cells, characterized by the presence of a large amount of mitochondria. The postoperative course was uneventful, and the patient experienced a progressive regression of Cushing-related symptoms. Periodical follow-ups with MRI and cortisol dosage are required due to the neoplasm's uncertain malignant potential. Considerations on the diagnosis, pathology findings, clinical remarks, and interventions are made.

## 1. Introduction

Adrenocortical oncocytic neoplasms (ACONs) are an epithelial subtype of adrenal tumours, firstly reported by Kakimoto et al. in 1986 [1]. Oncocytomas have distinctive histopathological features, as they are characterized by large cells with a wide granular eosinophilic cytoplasm, related to numerous swollen mitochondria [2, 3]. The adrenal gland is a very uncommon location for oncocytomas, which are usually found in different sites, such as kidneys, thyroid, parathyroid, pituitary, and salivary glands [3, 4]. These tumours are usually incidentalomas, unwittingly detected during radiological investigations performed for other medical reasons. Nevertheless, up to 35% are capable of synthesizing hormones such as aldosterone and cortisol, originating visible clinical manifestations [5]. Indeed, the virilisation and feminization syndromes due to sexual hormone hypersecretion are well described in the literature, as well as Cushing's and Conn's syndromes due to primary hypercortisolism and hyperaldosteronism, respectively. However, adrenocortical oncocytomas generally do not have particular radiological features but large dimensions for which the diagnosis is rarely made before surgery and pathology examination [6–8]. We, hereby, report a case of a 27-year-old woman undergoing left laparoscopic adrenalectomy at our institution, for endogenous Cushing's syndrome, due to an adrenocortical mass, which was also an oncocytoma of uncertain malignant potential.

## 2. Case Report

### 2.1. Clinical Presentation, Diagnosis, and Treatment

A 27-year-old Caucasian woman was referred to our Emergency Department due to the acute onset of severe hypertension (190/110). She reported one year of insomnia caused by recurring sleep hyperhidrosis. Apart from that, she had neither relevant medical history nor any previous surgical history. She was a regular cigarette smoker, with a body mass index of 21, who followed a vegan diet. Physical examination revealed a full-moon face, a mild buffalo hump, and a light hypotrophy of proximal limb muscles. In addition, dermatologic signs showed thin skin with the tendency to bruise, purple striae, thickened hair, and facial seborrheic dermatitis. There were no signs of virilisation nor did she report menstrual irregularities. Initially, hormonal studies showed a slightly elevated basal cortisol level (21.4 mg/dL, reference range 3.7–19.4 mg/dL); however, D4-androstenedione, progesterone, aldosterone, testosterone, and adrenaline urinary catabolites levels were normal. After performing an overnight suppression test with 1 mg of dexamethasone, basal serum cortisol was increased to 19.90 *μ*g/dL. Hormonal test results are summarized in Tables 1 and 2. An abdominal ultrasound was performed, highlighting a bilateral adrenal gland hypertrophy. Magnetic resonance imaging (MRI) with paramagnetic contrast showed a 23 mm nodular mass of the left adrenal gland cortex with regular and well-defined margins and early arterial postcontrast enhancement. Following the diagnosis of ACTH-independent Cushing's syndrome, the patient was referred for surgery. The patient underwent laparoscopic left adrenalectomy: firstly, a medial to lateral approach and no-touch technique was chosen to dissect and divide the left adrenal vein. There was no intraoperative rebound and no need for blood arterial pressure adjustment. There were no postoperative complications, and the young woman was sent home on the second postoperative day following ACTH and cortisol blood determination as well as 24 h urinary free cortisol testing.

### 2.2. Pathology Examination

**Figure 1:**
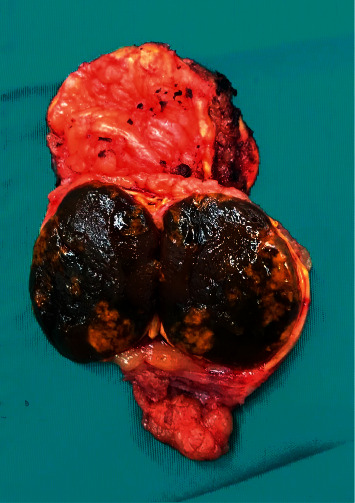
Macroscopic specimen: tan-brown neoplasm with regular and smooth borders largely replacing the normal adrenal gland parenchyma, which is recognizable at the margin of the specimen.

**Figure 2:**
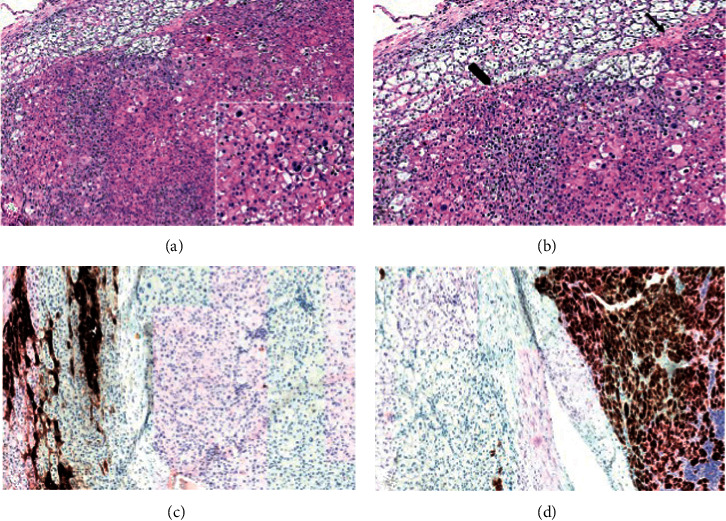
Histopathological features and immunohistochemical findings: (a) encapsulated diffuse proliferation of large cells with focal capsular invasion. Inset: the cells show pleomorphic nuclei, prominent nucleoli, and wide eosinophilic and granular cytoplasm. (b) At higher magnification, capsular invasion is shown in detail (the arrow signs a thin fibrous capsule dividing the neoplasm from the cortex of the adrenal gland, while the thick arrow shows the focal infiltration of the adrenal parenchyma by the tumour). (c) The neoplastic cells resulted negative for chromogranin A (positive internal control: adrenal medulla). (d) The neoplastic cells resulted strongly and diffusely positive for inhibin A (internal control: adrenal cortex, mild and sparse positivity). (a, b) Haematoxylin-eosin stain. (c) Chromogranin A. (d) Inhibin A. Original magnification: (a) 40x, inset 200x; (b) 200x, (c, d) 100x.

**Figure 3:**
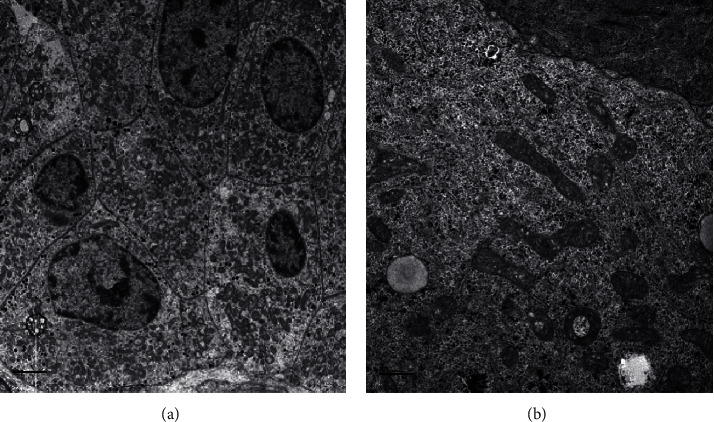
Electron microscopy findings: (a) electron micrographs show adrenal oncocytoma cells, characterized by the presence of large amount of mitochondria (scale bar represents 2 *μ*m). (b) high magnification displays ultrastructural details of crista-type mitochondria (scale bar represents 0.2 *μ*m).

The surgical specimen underwent histopathological examination: it consisted of adipose tissue containing a tan-brown neoplasm, with regular and smooth margins, of 2.7 × 2 cm in size, largely replacing the normal adrenal gland parenchyma (Figure 1). Microscopically, the nodule was composed of more than 90% of an encapsulated proliferation of oncocytes (cells with large eosinophilic and granular cytoplasm, pleomorphic nuclei, and prominent nucleoli) in a diffuse architectural pattern (Figure 2(a) and inset). The tumour only focally showed capsular infiltration (Figures 2(a) and 2(b)). Immunohistochemical tests revealed that the tumour cells were negative for chromogranin A (Figure 2(c)) and positive for inhibin A, a specific marker of adrenocortical origin (Figure 2(d)). Specific mitochondrial immunohistochemical techniques (mES-13; SDHA/SDHB) would have been helpful to prove that the eosinophilic aspect of the cells are indeed mitochondria accumulated in the cytoplasm, but unfortunately, we do not have it available. Electron micrographs confirmed adrenal oncocytoma cells, characterized by the presence of a large amount of mitochondria (Figure 3). Although neither necrosis nor vascular invasion was detected, one mitosis per 50 high-power fields was counted, thus resulting in a Ki-67 proliferation index lower than 5%. Based on the Lin–Weiss–Bisceglia (LWB) grading scale—reported by the WHO for oncocytic neoplasm of the adrenal gland [9]—a final diagnosis of oncocytoma of uncertain malignant potential was established.

### 2.3. Postoperative Outcome

Postoperatively, the patient began glucocorticoid and mineralocorticoid replacement therapies with cortisone 21-acetate 18, 75 mg per day, which were gradually tapered. One month after surgery, the patient had already experienced a progressive regression of Cushing-related symptoms and a relative normalization of blood pressure levels, as confirmed by a blood pressure Holter. She was advised to undergo quarterly ACTH cortisol dosage and referred for follow-up to our oncological team.

## 3. Discussion

ACONs are a rare epithelial subtype of adrenal tumours firstly reported by Kakimoto in 1986 [1]. More than 2/3 of ACONs are nonfunctioning or clinically silent. Functioning ACONs are reported with a frequency of 17–34%, of which less than 6% are cortisol-secreting. Moreover, although 41% of ACONs are of uncertain malignant potential, the functioning forms of these range between 22 and 29% [5, 8].

While renal oncocytomas have radiological features suggesting preoperative diagnosis, such as the central scarring, the same is not true for adrenal oncocytomas. Benign adrenal oncocytomas may be distinguished from lipid-rich adenomas on CT scan due to the fact that the latter show low attenuation on CT scan (<10 Hounsfield-HU), whereas the malignant oncocytic neoplasm can demonstrate features similar to adrenocortical carcinomas (ACCs), including larger size, internal necrosis, and lower percentage enhancement washout [10].

An ultrasound-guided biopsy is safe but rarely performed as it is frequently inconclusive and is difficult to use for the discrimination between kidney-originated tumours and adrenal tumours [11, 12]. Indeed, in all reported cases of ACONs, the diagnosis was always made at the histopathological examination. Immunohistochemical tests show positivity for inhibin A (69%), melan-A (85%), synaptophysin (74%), vimentin (80%), mitochondrial antibody mES-13 (100%), calretinin (78%), neuron-specific enolase (94%), and cytokeratin AE1/AE3 (52%); they show negativity for chromogranin A (91%) and S100 protein (96%) [5].

In the last edition of the WHO classification of endocrine neoplasms, the LWB-specific criteria was reported in order to classify ACONs as malignant when at least one major criterion was present, such as a high mitotic rate (>5 mitoses per 50 high-power fields), atypical mitoses, and venous invasion. ACONs of uncertain malignant potential are classified as such in the presence at least one of the minor criteria: large size (more than 10 cm), necrosis, and capsular and sinusoidal invasion [8, 13, 14]. In the case at hand, we found a minimal capsular invasion, and the neoplasm was classified as of uncertain malignant potential. The reticulin algorithm, reported by Volante et al. [15], was not used because although useful in discriminating adenomas from carcinomas, it does not determine the eventual malignant potential of adenomas with uncertain malignant potential as considering mitosis >5/10 HPF without taking account of features such as necrosis or vascular invasion. The case hereby reported had a Weiss score <3, and the presence of a disrupted reticulin framework would not be sufficient to be classified as malignant.

ACONs preferably arise from the left gland, with an M/F ratio of 1 : 2 and an average age of 44. The ACON reported retains an uncertain malignant potential since the capsule was focally infiltrated, but it was certainly a smaller lesion (2.7 × 2 cm) when compared to the average size of 10 cm for the commonly found borderline ACONs.

Prognostic factors still need to be validated because of the low number of patients reported in literature. However, males appear to be more frequently associated with malignant ACONs, whereas younger patients are more commonly affected by smaller benign ACONs compared to borderline and malignant ones [5].

Currently, ACONs of uncertain malignant potential appear to have an 88% probability of a 5-year survival rate, with only two recurrences reported in literature out of 57 cases. Although malignant potential is classified by the LWB criteria, there are still no formal guidelines regulating the follow-up. Surely, borderline and malignant ACONs should undergo periodic MRI/CT scan. Ipsilateral adrenalectomy is usually indicated for the uncertain nature of the mass and to resolve the concomitant symptomatology. Laparoscopy seems to achieve similar rates of R0 resection of open surgery for ENSAT I and II ACC and for masses smaller than 10 cm. Laparoscopic adrenalectomy is feasible, safe, and offers all the advantages of the minimally invasive surgical technique [16–19].

## 4. Conclusions

Functioning and cortisol-secreting ACONs are very rare entities, but they can certainly be responsible of ACTH-independent Cushing's syndrome. Surgical excision is indicated, and the laparoscopic approach appears to be feasible and safe in most instances. Since borderline ACONs are of uncertain malignant potential, a proper follow-up is warranted.

## Figures and Tables

**Table 1:** Laboratory tests.

		Before hospitalization	First visit	Overnight suppression test	2 days postsurgery	A month later surgery	Normal range
Plasma sodium	mEq/L	146	143		137	141	136–145
Plasma potassium	mEq/L	5.10	4.30		4.50	4.50	3.50–5.10
Plasma basal cortisol	*μ*g/dl	**28.4**	21.40	19.90	1.10	0.9	4.4–22.0
Plasma ACTH	pg/ml	**<5**	**<5**		<**5**	**<5**	0.0–46.00
24 h urinary free cortisol	mcg/24 h	**434.70**	**356.4**		2.5		0–100
24 h urinary sodium	mMol/24 h	78.30	**250.80**				40–220
24 h urinary potassium	mMol/24 h	75.60	**132.00**				25–125

Laboratory tests are conducted to determine the etiology of Cushing's syndrome showed modest increase in basal cortisol with abolition of its circadian rhythm and ACTH levels constantly and markedly suppressed. Bold values represent lab tests out of range and the ones useful in diagnosing ATCH-independent Cushing's syndrome.

**Table 2:** Other hormonal tests.

		Patient	Normal range
Metanephrine	mcg/24 h	292.00	64-303
Normetanephrine	mcg/24 h	345.00	163-530
Urinary aldosterone	mcg/24 h	<9.7	2.5–20
DHEA-S	mcg/dl	**15.20**	18.00–391
Total testosterone	ng/dl	8.20	13.84–53.35
Delta-4 androstenedione	ng/ml	0.56	0.3–3.3
Thyroid stimulating hormone	uU/ml	1.35	0.50–6.5
Free T3	pg/ml	2.88	1.3–3.6
Free T4	pg/ml	1.30	0.8–2.0
FSH	ng/ml	5.01	5.00–35.0
Prolactin	ng/ml	24.10	5.00–25
LH	mIU/ml	4.99	
17-Beta estradiol	pg/ml	37.86	
Progesterone	ng/ml	1.47	

Bold values are the ones out of range.
